# *Mycobacterium tuberculosis* Thymidylyltransferase RmlA Is Negatively Regulated by Ser/Thr Protein Kinase PknB

**DOI:** 10.3389/fmicb.2021.643951

**Published:** 2021-03-31

**Authors:** Dehui Qu, Xiaohui Zhao, Yao Sun, Fan-Lin Wu, Sheng-Ce Tao

**Affiliations:** ^1^Key Laboratory of Molecular Module-Based Breeding of High Yield and Abiotic Resistant Plants in Universities of Shandong, School of Agriculture, Ludong University, Yantai, China; ^2^State Key Laboratory of Microbial Metabolism and School of Life Sciences and Biotechnology, Shanghai Jiao Tong University, Shanghai, China; ^3^Key Laboratory of Systems Biomedicine (Ministry of Education), Shanghai Center for Systems Biomedicine, Shanghai Jiao Tong University, Shanghai, China

**Keywords:** PknB, *Mycobacterium tuberculosis*, RmlA, phosphorylation, cell wall

## Abstract

Ser/Thr phosphorylation by serine/threonine protein kinases (STPKs) plays significant roles in molecular regulation, which allows *Mycobacteria* to adapt their cell wall structure in response to the environment changes. Identifying direct targets of STPKs and determining their activities are therefore critical to revealing their function in *Mycobacteria*, for example, in cell wall formation and virulence. Herein, we reported that RmlA, a crucial L-rhamnose biosynthesis enzyme, is a substrate of STPK PknB in *Mycobacterium tuberculosis* (*M. tuberculosis*). Mass spectrometry analysis revealed that RmlA is phosphorylated at Thr-12, Thr-54, Thr-197, and Thr-12 is located close to the catalytic triad of RmlA. Biochemical and phenotypic analysis of two RmlA mutants, T12A/T12D, showed that their activities were reduced, and cell wall formation was negatively affected. Moreover, virulence of RmlA T12D mutant was attenuated in a macrophage model. Overall, these results provide the first evidence for the role of PknB-dependent RmlA phosphorylation in regulating cell wall formation in *Mycobacteria*, with significant implications for pathogenicity.

## Introduction

Tuberculosis is still a leading challenge for public health. An estimated 10 million people fell ill with TB in 2019, and it is one of the top 10 causes of death worldwide (WHO, 2020). *Mycobacterium tuberculosis* (*M. tuberculosis*), the causative pathogen of tuberculosis, has developed a complex mechanism to combating immune reaction (Flynn and Chan, 2003). The unique composition of *M. tuberculosis* cell wall is a key factor for its survival in the infected host. Although there are a variety of studies about cell wall components and functional analysis in recent years, little is known about the mechanisms of its cell wall components in response to environmental stimulation. Therefore, revealing the function of the cell wall components plays a significant role in understanding the physiology of *M. tuberculosis*.

**FIGURE 1:**
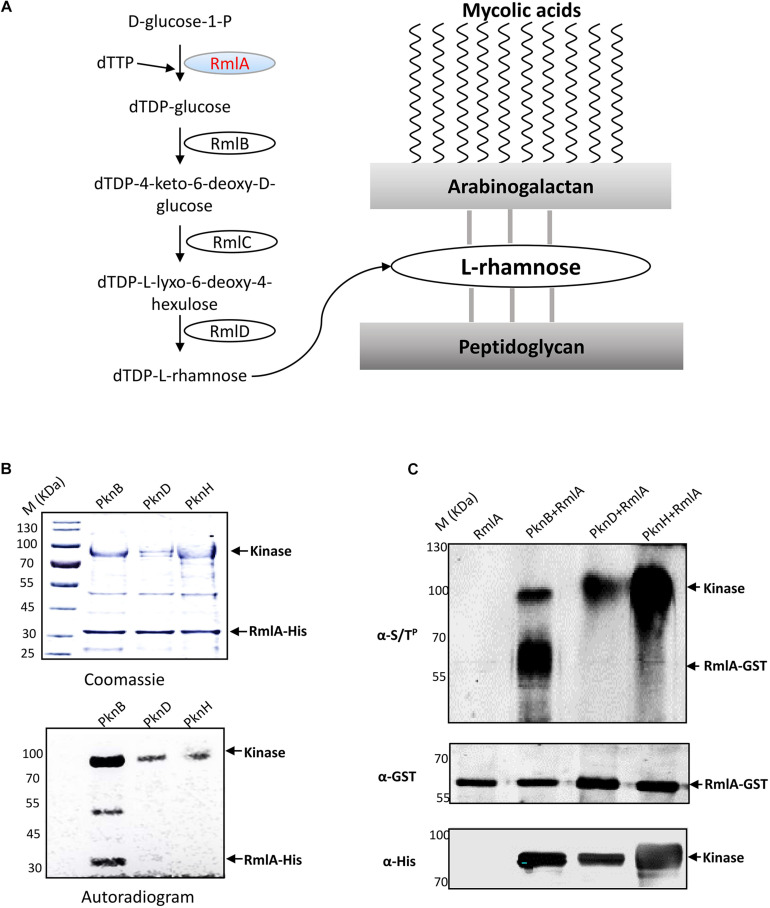
RmlA, an important enzyme for L-rhamnose formation, is phosphorylated by PknB. **(A)** The biosynthesis pathway of cell wall linker-L-rhamnose. dTDP-rhamnose is the precursor of L-rhamnose. The biosynthetic pathway of dTDP-rhamnose consists of four steps from a-D-glucose-1-phosphate and TTP to dTDP-rhamnose. **(B)** *In vitro* phosphorylation of *M. tuberculosis* RmlA by STPKs. The three recombinant *M. tuberculosis* STPKs (PknB/D/H) were expressed and purified as His-tagged fusions and incubated with purified His-tagged RmlA and [γ−^32^P] ATP. Samples were separated by SDS-PAGE, stained with Coomassie blue (upper panel), and visualized by autoradiography after overnight exposure to a film (lower panel). **(C)** In *E. coli* phosphorylation of *M. tuberculosis* RmlA by STPKs. *rmlA* gene was cloned into pDEST15 vector with a GST tag using the Gateway system, whereas the kinases (*pknB*, *pknD*, and *pknH*) were cloned into pET28a vector with His tag. The two plasmids were cotransformed into *E. coli* BL21 (DE3), and the RmlA and kinases were enriched with GST and Ni^2+^ agarose beads, respectively. After immunoblotting, the phosphorylated signals were detected with an antithreonine phosphorylation antibody.

The cell wall of *M. tuberculosis* is complex, which contains three important components including peptidoglycan, arabinogalactan, and mycolic acids. L-Rhamnose links the mycolic acid layer to the inner peptidoglycan layer (Giraud and Naismith, 2000). The structural integrity of the cell wall based on the linking of L-rhamnose plays an important role in maintaining *M. tuberculosis* viability. dTDP-L-rhamnose is synthesized from glucose-1-phosphate (G1P) and deoxy-thymidine triphosphate (dTTP) by four enzymes, including glucose-1-phosphate thymidylyltransferase (RmlA), dTDP-D-glucose 4,6-dehydratase (RmlB), dTDP-6-deoxy-D-xylo-4-hexulose-3,5-epimerase (RmlC), and dTDP-6-deoxy-L-lyxo-4-hexulose reductase (RmlD) (Giraud and Naismith, 2000; Ma et al., 2001; Figure 1A). RmlA is the rate-limiting enzyme of this pathway. It is reported that RmlA is essential for *M. tuberculosis* growth *in vitro* and survival during infection *in vivo* (Sassetti et al., 2003). Furthermore, Qu et al. (2007) showed that *rmlA* knockout could affect cell growth and morphology of *Mycobacterium smegmatis* (*M. smegmatis*). The attachment of these two layers by L-rhamnose in *M. tuberculosis* is unique, which does not appear in mammalian and most of the bacteria polysaccharides. Thus, RmlA represents an attracting potential target for development of antimycobacterial drugs.

It is well known that Ser/Thr phosphorylation plays a central role in the mechanism for sensing and responding to external signals. As in eukaryotes, the STPKs play critical roles in environmental sensing and downstream signaling. However, in *M. tuberculosis*, the 11 eukaryotic-like serine/threonine protein kinases (STPKs) are usually involved in the regulation of metabolic processes (O’Hare et al., 2008; Rieck et al., 2017), transportation (Hatzios et al., 2013), cell division (Nagarajan et al., 2015; Sharma et al., 2016), or virulence (Molle et al., 2008; Khan et al., 2010). Therefore, regulation through ser/thr phosphorylation plays a key role in the pathology of *Mycobacteria*. PknB, a well-known *M. tuberculosis* STPK, is believed to regulate cell wall biosynthesis and cell division (Banu et al., 2010). The extracellular domain of PknB binds peptidoglycan-derived muropeptides for its localization (Roumestand et al., 2011), and several phosphorylated substrates have been identified, such as SahH (Singhal et al., 2013), InhA (Vilcheze and Jacobs, 2014; Shaw et al., 2017), KasB (Vilcheze et al., 2014), GlmU (Parikh et al., 2009), MmpL3 (Le et al., 2020), CwIM (Turapov et al., 2018), and Ef-Tu (Sajid et al., 2011), in participating in cell metabolic, cell wall synthesis, transcription, and translation processes.

According to the critical importance of L-rhamnose in *M. tuberculosis* cell wall formation, the rhamnose synthesis pathway must be tightly regulated. It was reported that PrkA, which is a homolog of PknB, interacts with glucose-1-phosphate thymidylyltransferase (similar to RmlA) using affinity chromatography and proteomic approaches in *Listeria monocytogenes* (Lima et al., 2011). Moreover, in our previous study, we also found that some STPKs, especially PknB, strongly interact with RmlA (Wu et al., 2017). However, the regulation mechanism of RmlA is still largely unknown. Thus, the relationship of PknB with RmlA was explored. In this study, we found that PknB can effectively phosphorylate RmlA and down-regulate its activity. We then proposed a three-dimensional (3D) model to explain the inhibition of PknB phosphorylation on the activity of RmlA. More importantly, the mutations of the phosphorylation sites have a series of phenotypic effects, e.g., slower cell growth, weaker acid staining, reduced biofilm formation, and sensitive to external stimulus and in macrophage survival. Based on these data, we revealed a novel mechanism of *M. smegmatis*, a model strain for *M. tuberculosis*, in cell wall regulation; i.e., PknB phosphorylates RmlA and inhibits its activity. Our study provides the first evidence-based link between STPKs and L-rhamnose biosynthesis pathway.

## Materials and Methods

### Bacterial Strains, Plasmids, and Growth Conditions

Bacteria strains used for cloning and expression of recombinant proteins were *Escherichia coli Trans* 5α (Transgen Biotech, Beijing, China) and *E. coli* BL21 (DE3) (Transgen Biotech, Beijing, China), and they were grown in LB medium at 37°C. Ampicillin (100 μg/mL), hygromycin (150 μg/mL), or kanamycin (50 μg/mL) was added to the medium. *M. smegmatis* mc^2^155 strain was grown on Middlebrook 7H10 agar (BD, NJ, United States) plates or in Middlebrook 7H9 medium (BD, NJ, United States), added with kanamycin (25 μg/mL) or hygromycin (50 μg/mL) (Supplementary Table 1). The plasmids used in this study are listed in Supplementary Table 1.

### Cloning, Purification of Recombinant RmlA, RmlA Mutants, and STPK Proteins in *Escherichia coli*

The *rmlA* gene and kinases (*pknB*, *pknD*, and *pknH*) genes from *M. tuberculosis* were cloned to pDEST15 or pET28a (Supplementary Table 1). All constructs were verified by DNA sequencing. Recombinant RmlA and STPKs proteins were overexpressed in *E. coli* BL21 (DE3), and the strains were inoculated into 100 mL LB liquid medium supplemented with kanamycin at 37°C until the OD_600_ to 0.6–0.8. IPTG was added with the final concentration at 1 mM, and growth was continued at 25°C for 6 h. Protein purification assay was performed as previously described (Wu et al., 2017). Cells were then harvested by centrifugation 6,000 *g* for 5 min, resuspended in lysis buffer (50 mM Tris–HCl, pH 7.5; 100 mM NaCl, 1 mM EGTA; 0.5 mM PMSF; one Roche protease inhibitor cocktail). Bacteria were disrupted in an ultrahigh pressure homogenizer (Yonglian, Shanghai, China), and the lysis was centrifuged for 10 min at 10,000 *g* at 4°C. The supernatant was collected, and Ni^2+^-agarose beads were added. After 2 h, the beads were washed with wash buffer (50 mM Tris–HCl, pH 7.5; 300 mM NaCl; 20 mM imidazole) for three times. Lastly, beads were eluted with elution buffer (50 mM Tris–HCl, pH 7.5; 100 mM NaCl; 300 mM imidazole). The purified proteins were stored at −80°C until use.

### Coexpression of RmlA and STPK in *Escherichia coli*

The constructed plasmids of RmlA-pDEST15 and STPK-pET28a were mixing together with mass ratio of 1:1. Then, they were cotransformed into the *E. coli* BL21 (DE3) with the addition of kanamycin (50 μg/mL) and ampicillin (100 μg/mL). A single clone was picked up and inoculated to 5 mL LB medium with kanamycin and ampicillin and cultured overnight at 37°C, 200 rpm. The bacteria was then inoculated to 100 mL LB medium with kanamycin and ampicillin at a dilution of 1:100 and cultured for approximately 3 h to OD_600_ = 0.6–0.8. Then, 50 μL IPTG (1 M) was added to the medium, followed by culture at 25°C for 6 h. The cells were harvested by centrifugation at 6,000 *g* for 5 min. The cells were resuspended with lysis buffer and were broken down with an ultrahigh pressure homogenizer. The proteins were then purified with GST and Ni^2+^ agarose beads separately and quantified with a silver staining kit (Beyotime, Nantong, China). After separation with sodium dodecyl sulfate–polyacrylamide gel electrophoresis (SDS-PAGE) gel and transferred to a nitrocellulose membrane, the membrane was saturated with 5% skim milk and probed with either a rabbit anti-His antibodies (Sigma–Aldrich, St. Louis, MO, United States), a rabbit anti-GST antibodies (Sigma–Aldrich, St. Louis, MO, United States), or an anti–serine/threonine phosphorylation antibodies (dilution, 1:1,000) (Abcam, Cambridge, MA, United States). After washing, the membrane was incubated with a secondary goat–anti-rabbit antibody (LI-COR, NE, United States) and scanned using the Odyssey system (LI-COR) according to the manufacturer’s instructions.

### *In vitro* Kinase Assay

*In vitro* phosphorylation assay was performed as described in a previous study (Shakir et al., 2010), with some modifications. Briefly, 5 μg of wild-type (WT) RmlA was added to 25 μL of kinase buffer (50 mM Tris–HCl, pH 7.5; 1 mM DTT; 10 mM MgCl_2_; and 50 mM NaCl) together with 200 μCi/mL of [γ-^32^P]ATP (PerkinElmer Life Sciences, 3,000 Ci/mmol), and 4 μg of kinase for 2 h at 37°C. The reactions were stopped by the addition of SDS loading buffer, and the sample was heated at 95°C for 5 min. After electrophoresis, the radioactive proteins were visualized by autoradiography using direct exposure to films.

### Mass Spectrometry Analysis

Purified GST-tagged RmlA and His-tagged PknB kinase from the *E. coli* were subjected to mass spectrometry analysis after kinase assay *in vitro*. And the mass spectrometric analyses were performed as reported previously (Leiba et al., 2013).

### Analysis of RmlA Structure

The RmlA protein structure of *Pseudomonas aeruginosa* (1g2v.pdb) was download from the PDB database^1^. The 3D images of the crystal structure of RmlA were shown using Pymol (Jenkinson and McGill, 2012).

### Enzymatic Assays

The colorimetric assay for RmlA enzyme activity was performed as described (Sha et al., 2012). Briefly, 5 μg of purified RmlA was added to 50 μL of reaction buffer (50 mM Tris–HCl, pH 7.5; 1 mM DTT; and 5 mM MgCl_2_) with 0.2 mM dTTP, 1 mM D-Glc-1-P, and 0.04 unit of *Saccharomyces cerevisiae* pyrophosphatase (Sigma–Aldrich, St. Louis, MO, United States) for 30 min at 37°C. The reactions were stopped by the addition of 50 μL malachite green reagent [0.03% (wt/vol), 0.2% (wt/vol) ammonium molybdate, and 0.05% (vol/vol) Triton X-100 in 0.7 N HCl] at 37°C for 5 min. The signal was detected for the absorbance at 630 nm by a microplate reader.

### Cloning, Overexpression of RmlA in *Mycobacterium smegmatis*

*Mycobacterium tuberculosis rmlA* and mutated genes were cloned into the shuttle vector pMV261 with His tag using the primers listed in Supplementary Table 2. The resulting constructs pMV261-RmlA and mutants were introduced by electroporation into *M. smegmatis*. Transformants were grown in the 7H10 medium with 25 μg/mL kanamycin. A single clone was picked up and inoculated to 100 mL 7H9 medium added with kanamycin, cultured for 48 h at 37°C, 200 rpm. The cells were harvested by centrifugation at 6,000 *g* for 5 min. Resuspended with lysis buffer (50 mM Tris–HCl, pH 7.5; 100 mM NaCl; 1 mM EGTA) and broken down with an ultrahigh pressure homogenizer. The proteins were then purified with Ni^2+^ agarose beads and quantified with Coomassie brilliant blue staining. The purified recombinant proteins were used for immunoblotting using the antithreonine phosphorylation antibody.

### PknB Conditional Knockdown in *Mycobacterium smegmatis* Strain

To test the *in vivo* effect of PknB on RmlA, we adopted a published *Mycobacteria* CRISPRi system to conditional knockdown PknB in *M. smegmatis* (Rock et al., 2017). Briefly, the four sgRNA scaffolds were designed with two unique *Bsm*BI restriction sites immediately 5′ to the sgRNA scaffold sequence. Complementary sgRNA targeting oligos (N20–25) were then annealed and ligated into the *Bsm*BI-digested CRISPRi vector backbone (PLJR962). sgRNA targeting sequences are listed in Supplementary Table 2. The constructed plasmid of sgRNA-dCas9_sth1_ was electronically transformed to *M. smegmatis*, and cultured on 7H10 medium (25 μg/mL kanamycin). To detect the mRNA level of *pknB* expression, cultures were grown to log phase, and 100 ng/mL ATc was added to induce the functionalization of CRISPRi. Targeted knockdown was allowed to proceed for 24 h. Two OD_600_ equivalents of cells from each culture were harvested by centrifugation, and RNAs were purified with RNA extraction kit (Tiangen, Beijing, China). cDNA was prepared with manufacturer instructions (Promega, Madison, WI, United States). cDNA levels were then quantified by quantitative real-time polymerase chain reaction (qRT-PCR) using Universal SYBR Green mix (Thermo, MA, United States). Signals were normalized to the housekeeping *sigA* transcript for *M. smegmatis* and quantified by the ^ΔΔ^Ct method. Error bars are 95% confidence intervals of three technical replicates.

### Immunoprecipitation and Immunoblotting

The constructed plasmid of sgRNA-dCas9_sth1_ was electronically transformed to *M. smegmatis* and cultured on 7H10 medium (25 μg/mL kanamycin). Then, the transformants were transformed to 100 mL 7H9 medium (25 μg/mL kanamycin) with 100 ng/mL ATc to induce the functionalization of CRISPRi, whereas the control was set without ATc induction. After 48-h induction, cells were collected and washed by phosphate-buffered saline (PBS) under 8,000 *g* for 5 min. The cells were resuspended with PBS and broken down with an ultrahigh pressure homogenizer, and the resulted extract was centrifuged for 15 min, 10,000 *g* at 4°C. The supernatant was collected and added with 10 μL anti-RmlA antibody (anti-rabbit) (Abmart, Shanghai, China), incubated overnight at 4°C with gentle shaking. Then, 50 μL Protein G-agarose beads were added to pull down the antibody with incubation for 2 h at 4°C. After incubation, the beads were collected by centrifugation (3,000 rpm/min, 5 min) and washed by PBS for five times. The beads were resuspended with 100 μL 1 × PBS and 6 × SDS loading buffer (Beyotime Biotechnology, Nantong, China). After 10 min of denaturation at 95°C, the samples were subjected to electrophoresis and Western blot detection with antithreonine phosphorylation and anti-RmlA antibodies.

### Generation of *rmlA* Mutants in *Mycobacterium smegmatis*

A two-step homologous recombination strategy was used to disrupt the *M. smegmatis rmlA* gene at its native locus (Stephan et al., 2005). Two vectors pMZ(+), the gene manipulating vector without any mycobacterial origin of replication, and pCMG50, the marker gene cassette-hyg-containing vector, were used for this purpose. Approximately 800 bp upstream and downstream sequence of *rfpA* in *M. smegmatis* and *rmlA* mutants genes (6 × His) of *M. tuberculosis* were PCR amplified (Supplementary Table 2). Through nest PCR, the three PCR fragments were connected and cloned into the vector of pMZ(+) with the restriction sites *Sac*I and *Kpn*I to generate the pMZ(+)-RmlA. Finally, a 1.5 kb *Spe*I fragment carrying the Hyg gene was excised from pCMG50 and inserted into pMZ(+)-RmlA to generate the suicide plasmid pMZ(+)-RmlA-Hyg. pMZ(+)-RmlA-Hyg was electroporated into competent cells of *M. smegmatis* mc^2^155 and plated onto Middlebrook 7H10 agar plates supplemented with hygromycin B (50 μg/mL). The colonies were isolated and identified with PCR validation.

### Mass Spectrometry Detection for the RmlA Activity *in vivo*

*Mycobacterium smegmatis*, *M. smegmatis-PknB KD*, and *M. smegmatis* RmlA mutants were grown in 7H9 medium at 37°C for 24 h. The strain pellet 20 mg was collected and resuspended in 5 mL ddH_2_O. The resuspended solution was boiled for 10 min with 40% methanol and 40% acetonitrile buffer mixture. Centrifugation 8,000 rpm was done to remove the protein and other undissolved substance. The supernatant was transferred to a new tube and freeze-dried overnight; 20 μL ddH_2_O and 80 μL methanol were added to resuspend it. The samples were performed with LC-HRMS as described previously (Vogliardi et al., 2011). The product of dTDP-D-Glc with molecular weight 563.0667 was extracted for analysis.

### Stress Assays

Minimum inhibitory concentrations (MICs) were determined using the broth microdilution method as previously described (Hatzios et al., 2013). For nitrosative stress, *M. smegmatis* strains were grown to mid–log phase and diluted to an OD_600_ of 0.1 in 7H9 acidified liquid medium (pH 5.5), and NaNO_2_ (0, 2, 4, 6, and 8 mM) were added. Then, the colony-forming units (CFUs) were determined after 2 days. For oxidative stress, *M. smegmatis* strains were prepared as above, but in non-acidified 7H9, medium was adjusted to an OD_600_ of 0.1; a series of concentration H_2_O_2_ (0, 1, 2, 4, and 6 mM) were included, and CFUs were determined after 12 h. All experiments were performed in triplicate and plating on 7H10 agar medium; the CFUs were counted after 2 days at 37°C.

### Acid-Fast Staining

The RmlA WT and mutants were grown to log phase, and 100 μL of culture was spread onto a glass slide. The slides were heated at 100°C for 2 min, dipped into 10% formalin for 30 min, dried, and stained using the TB Stain Kit K (BD, Carbolfuchsin staining) with manufacturer’s instruction.

### Biofilm Formation

The method for biofilm formation assay refers to a previous article (Ojha et al., 2005). For biofilm cultures grown on liquid medium, 10 mL of biofilm medium with a modified version of M63 in a 90 × 15-mm pvc Petri dish was inoculated with 10 μL of a saturated culture and incubated at 30°C without disturbance.

### Infection of Macrophage Cell

The infection assay for *M. smegmatis* was performed as described in a previous study (Wang et al., 2015), with some modifications. Briefly, THP-1 cells were differentiated to macrophages with 10 ng/mL PMA for 8 h. Then, the suspended *M. smegmatis* strains were infected with the THP-1 cells at a multiplicity of infection of 10:1. After 2-h infection, the cells were washed with PBS three times to exclude non-internalized bacteria and added with the fresh RPMI1640 medium supplemented with 10 μg/mL gentamicin to kill extracellular bacteria. The cells and the cell culture media for CFU counting or quantitative PCR at different time points were collected. Cells used for bacterial counting were lysed in 7H9 broth containing 0.05% SDS for 10 min. Three sets of serial 10-fold dilutions of the lysates from each time point were prepared in 0.05% Tween-80, and portions were plated on 7H10 agar plates. After 3 days, the number of bacteria in the plates with serial dilutions was counted.

## Results

### RmlA Is Phosphorylated by PknB

In our previous study, we found that PknB strongly binds to RmlA (Supplementary Figure 1). We speculated that RmlA was also the substrate of PknB. To test this, recombinant RmlA and STPKs were overexpressed and purified through His tag. According to the autophosphorylation assay, three kinases, i.e., PknB, PknD, and PknH, demonstrated high kinase activities (Supplementary Figure 2). Then, the phosphorylation assays were carried out with [γ-^32^P] ATP, RmlA, and these three kinases. After the kinase assay, the protein phosphorylation status was analyzed by autoradiography. The result showed that RmlA could be phosphorylated by PknB, whereas other kinases have little radioactive signal (Figure 1B). To further validate the kinase–substrate relationship between RmlA and PknB, GST-tagged RmlA and His-tagged kinases (PknB, PknD, and PknH) were coexpressed in *E. coli.* RmlA and the kinases were purified with GST and Ni^2+^ beads affinity, respectively. Following this, RmlA was separated by SDS-PAGE and analyzed by immunoblotting with anti-Ser/Thr phosphorylation antibody. The result indicated that RmlA is phosphorylated upon the overexpression of PknB, but not PknD and PknH (Figure 1C). These results suggest that PknB could phosphorylate RmlA *in vivo*. To further confirm the specific phosphorylation sites of RmlA by PknB, mass spectrometry analysis assay was performed. T12 (Table 1 and Supplementary Figure 3), as well as T54 and T197 were determined as the phosphorylation sites on RmlA, especially 80% phosphor-peptides had been found with T12.

**TABLE 1:** Phosphorylation sites of RmlA by PknB identified with mass spectrometry.

**Phospho peptides**	**Number of phospho peptides**	**Phospho- site**	***P* value**	**Percentage of phospho peptides**
GIILAGGSG**t**R	4	Thr-12	7.68 × 10^–8^	80% (4/5)
DIQLIT**t**PHDAP GFHR	4	Thr-54	5.25 × 10^–7^	33% (4/9)
GEYEI**t**EVNQV YLNQGR	2	Thr-197	2.37 × 10^–8^	33% (2/6)

### RmlA Is Mainly Phosphorylated by PknB With T12 Site in *Mycobacterium smegmatis*

**FIGURE 2:**
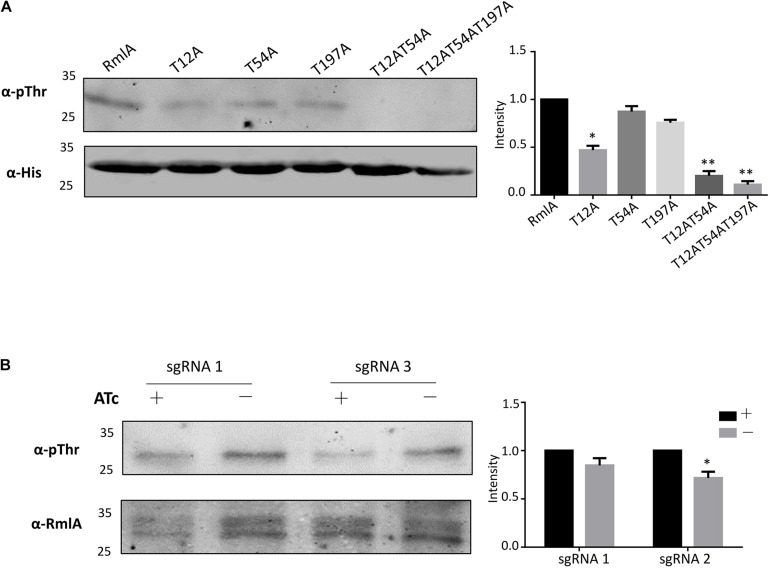
RmlA is mainly phosphorylated by PknB with T12 site. **(A)** Phosphorylation of RmlA and mutants in *M. smegmatis*. The WT and mutants of *rmlA* genes were cloned into pMV261 with His tag and incorporated into *M. smegmatis*. The proteins were purified with Ni^2+^ agarose beads. With Western blotting, phosphorylated signals were detected with the antithreonine phosphorylation antibody. The experiment was performed in triplicates (*n* = 3). Error bars indicate standard error of mean. Asterisk indicates significant difference (*P* < 0.05). **(B)** Phosphorylation of RmlA with the *pknB*-KD in *M. smegmatis*. The RmlA protein was purified from strains with sgRNA 1 and sgRNA 3 targeting, which have strong repression of PknB expression, using anti-RmlA antibody, and pull-down with protein G beads. Through Western blotting, phosphorylated signals were detected with the antithreonine phosphorylation antibody. The experiment was performed in triplicates (*n* = 3). Error bars indicate standard error of mean. Asterisk indicates significant difference (*P* < 0.05).

To further validate the identified phosphorylation sites *in vivo*, we used the model *Mycobacteria* strain *M. smegmatis*. The *M. tuberculosis* WT RmlA and the mutants (T12A, T54A, T197A, T12A/T54A, and T12A/T54A/T197A) with His tag were transformed to *M. smegmatis* and overexpressed. After affinity purification, the WT RmlA and RmlA mutants were subjected to Western blotting using the anti-Thr phosphorylation antibody. The result showed that significant loss of phosphorylation was observed for T12A, whereas there was a small change in T54A and T197A mutants (Figure 2A). Moreover, there was no significant phosphorylation signal in double mutant (T12A/T54A) and triple mutant (T12A/T54A/T197A) (Figure 2A). The results led to the possibility that the three sites (T12A, T54A, and T197A) all could be phosphorylated *in vivo*, and T12 represents the primary phosphorylation site, consistent with the fact that T12 was the most dominant phosphor-peptide identified by MS. To further test whether PknB is the primary STPK for phosphorylating RmlA, we constructed a *pknB* knockdown strain in *M. smegmatis*. Four sgRNA sequences were designed targeting the open reading frame of *pknB*; among them were two sgRNAs at the N terminal, one in the middle and another at the C terminal (Supplementary Figure 4A). And the most effective and available PAM sequences were chosen (Supplementary Figure 4B). The result showed that sgRNA 3 and sgRNA 1 could effectively repress the mRNA level of PknB to 1/10 and 1/3, respectively (Supplementary Figure 4C). Thus, sgRNA 1 and sgRNA 3 were then selected for conditional knockdown of PknB. After induction by ATc (anhydrotetracycline), RmlA was immune-precipitated with an anti-RmlA antibody. The phosphorylation signal was then monitored with the anti-Thr phosphorylation antibody. The most significant reduction of RmlA phosphorylation was observed when sgRNA 3 was applied (Figure 2B). These results indicated that PknB indeed is the key kinase for RmlA phosphorylating with T12 site in *M. smegmatis*.

### T12 Is in the Vicinity of RmlA Catalytic Center

**FIGURE 3:**
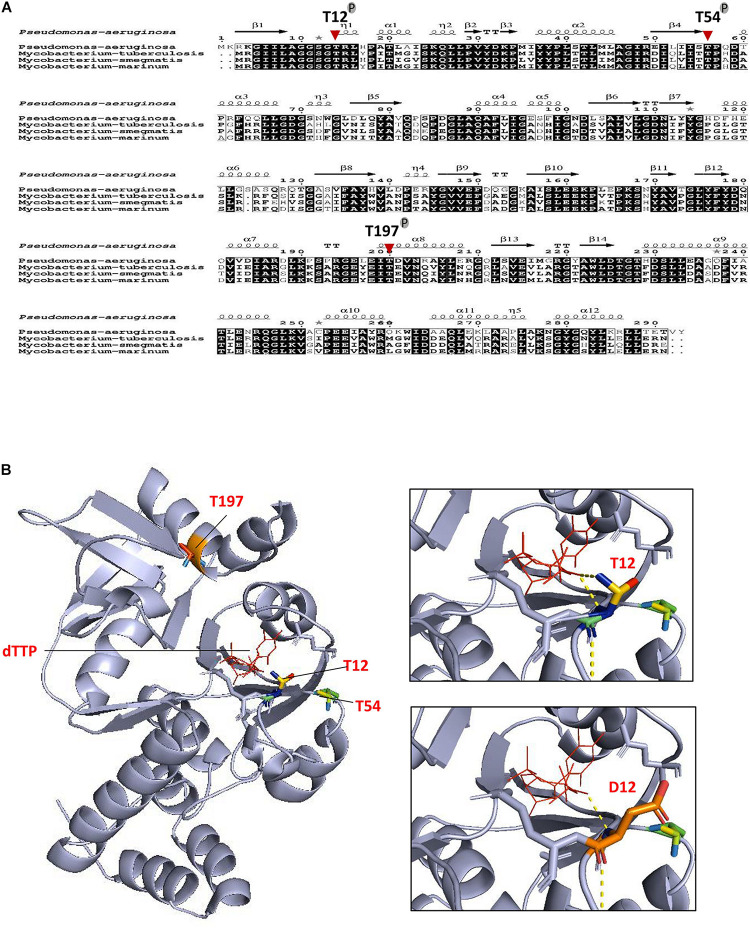
Conservation of phosphoacceptors in RmlA orthologs. **(A)** Multiple sequence alignment of RmlA sequences from *Pseudomonas aeruginosa* to mycobacterial species. The alignment was performed using ClustalW and Espript. Residues conserved in all species are shown in black boxes. The three phosphorylation sites of RmlA are indicated. Protein secondary element assignments are represented above the sequences. Numbering of amino acids corresponds to the RmlA protein from *P. aeruginosa*. **(B)** Localization of T12, T54, T197 phospho-sites in the three-dimensional structure of RmlA. Overall view (left panel) showing the RmlA monomer structure in ribbon representation with the α-helix and the β-sheet. Close-up view (right panel) indicates side chains of residues delineating the active site hydrophobic tunnel.

The 3D structure of *M. tuberculosis* RmlA has not been resolved yet. The structure of *P. aeruginosa* RmlA is already known, and the amino sequence of *M. tuberculosis* RmlA is highly conserved to that of *P. aeruginosa* RmlA (86%), as seen by the homolog site of *M. tuberculosis* RmlA T12, which corresponds to T14 of *P. aeruginosa* RmlA (Figure 3A). We then applied the structure of *P. aeruginosa* RmlA for mechanistic analysis. The active center of RmlA lies in a deep pocket formed by core and sugar-binding domains. dTTP locates in the active site, and the residues of G11, T12, and R13 are catalytically important (Figure 3B). T54 lies in the N-terminal of the β4 sheet, and T197 lies in the α8 helix. However, the RmlA active sites are located at the dimer–dimer interfaces, and crucial residues lie in the 10–24, 138–148, 160–162, and 224–232 loops, which are key in binding and orienting G1P and dTTP. Hence, the two phosphor sites, T54 and T197, are not close to the catalytic center and may not be able to significantly affect the activity of RmlA. Meanwhile, the flexible loop, in which T12 locates, is important for stabilizing the binding of dTTP, as the γ-phosphate forms a hydrogen bond with T12 (Figure 3B, right upper). Replacement of T12 by Asp, which mimics the phosphorylation, can possibly induce a perturbation with steric hindrance and electrostatic potential and also decrease the hydrogen bond (Figure 3B, right lower). Thus, the interference brought by the phosphorylation mimic might affect the enzymatic activity.

### T12 Site Is Crucial for RmlA Enzymatic Activity

**FIGURE 4:**
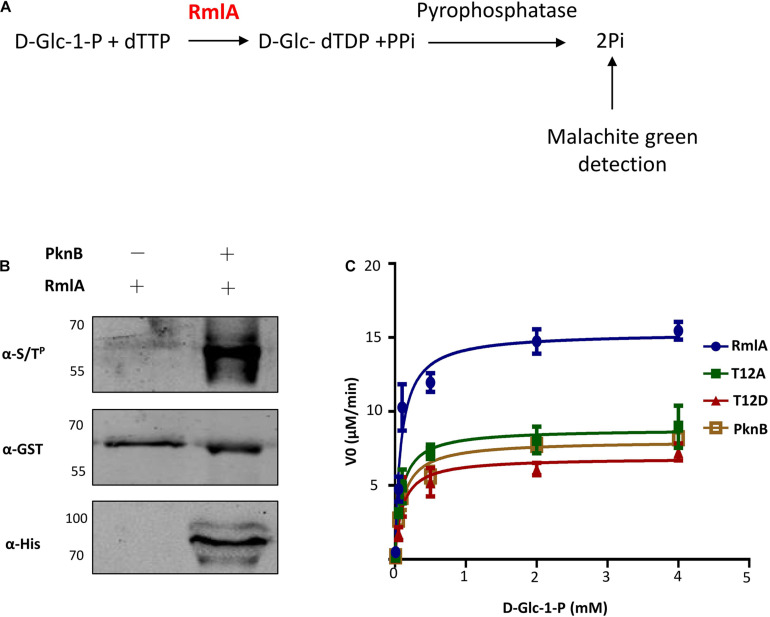
T12 of RmlA is a key residue for activity. **(A)** Schematic depicting the biochemical reaction catalyzed by RmlA. **(B)** Phosphorylation detecting with RmlA by PknB coexpression. **(C)** The activities of RmlA, RmlA-PknB coexpression, and its T12 mutants were determined based on Michaelis–Menten kinetics, representative of three. Five micrograms of purified RmlA protein was added to the 50 μL of enzyme reaction buffer [50 mM Tris (pH 7.5), 1 mM dithiothreitol, 5 mM MgCl_2_, 0.2 mM dTTP, and 1 mM D-Glc-1-P] and incubation at 37°C for 1 h.

To test whether the activity of *M. tuberculosis* RmlA could be affected by phosphorylation with PknB, a colorimetric assay was applied to assess the ability of RmlA to convert D-Glc-1-P and dTTP to D-Glc-dTDP and PPi, coupled with the conversion of PPi to Pi by malachite green detection (Figure 4A). The purified recombinant RmlA was phosphorylated by PknB when coexpressed in *E. coli* (Figure 4B). Meanwhile, the activity of RmlA was also affected by PknB coexpression (Figure 4C). Additionally, mutated RmlAs (T12A, T54A, T197A, T12A/T54A, and T12A/T54A/T197A), as well as the WT RmlA, were also purified and subjected for activity assessment. The results clearly demonstrated that T12 is the most critical site in terms of affecting the activity of RmlA (Supplementary Figure 5). Furthermore, we evaluated the influence of T12 mutants of phosphomimetic (aspartate) and phosphoablative (alanine) on the activity of RmlA. The results clearly demonstrated that these mutants indeed attenuate the activity of RmlA, which indicates the significant role of active site played to RmlA’s activity (Figure 4C). Statistically, the activity of T12A is higher than that of T12D. This indicates that the T12 site is indeed important for the activity of RmlA. These results are consistent with the structural analysis, which indicates that the introduction of Asp on T12 does influence the enzymatic activity of RmlA.

### *Mycobacterium tuberculosis* RmlA T12 Site Mutation Reduces Acid-Fast Staining and Biofilm Formation

To investigate the effects of phosphorylation on the conserved site of *M. tuberculosis* RmlA, T12 was endogenously replaced either with Ala or Asp. Homologous recombination method was used to transfer *M. tuberculosis* WT *rmlA*, T12A, and T12D mutant genes to replace the *M. smegmatis* intact *rfbA* gene (homologous to *M. tuberculosis* rmlA gene) (Supplementary Figure 6A). These recombinant strains contained a hyg cassette inserted between the gene of *rmlA* and *M.SMEG5982*. Then, the inserted genes including mutated *M. tuberculosis rmlA* gene and hyg cassette were verified with DNA electrophoresis, which has a 1,500-bp extension (Supplementary Figure 6B) compared with WT, and also with DNA sequencing (Supplementary Figure 6C). The protein expression was monitored with an anti-His antibody; comparable level of RmlA was observed among WT, T12A, and T12D (Supplementary Figure 6D). These results indicate that the Ala or Asp mutation does not change the protein level of RmlA in *M. smegmatis*, thus allowing analysis of the phenotypes with these mutations. The strains were then cultured and compared; the difference on growth curve indicates that the mutation on T12 of RmlA affects the growth of the two mutants (Supplementary Figure 6E).

**FIGURE 5:**
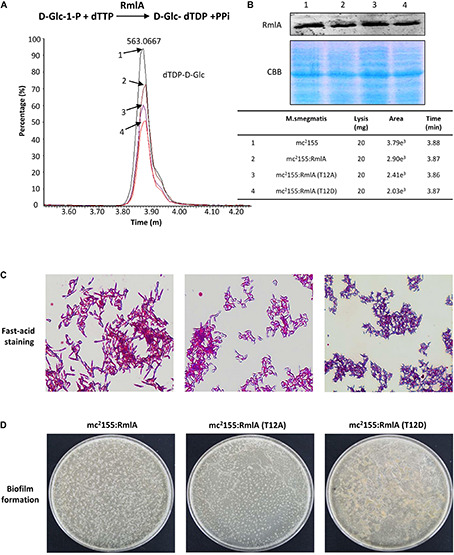
RmlA mutants affect cell morphology. **(A)** Detection of the activity of RmlA wild type and mutants. An RmlA activity assay was performed using D-Glc-1-P and dTTP as substrates. The major product of this reaction, dTDP-D-Glc, was measured by ultraperformance liquid chromatography and mass spectrometry. **(B)** Quantification and statistics analysis of the production of dTDP-D-Glc with wild type and mutants of RmlA. **(C)** Cultures were fixed on glass slides, and acid-fast staining was performed on the fixed smears using the BD Carbolfuchsin kit and detected with optical microscope. Magnification × 40. **(D)** The biofilm formation of wild type and mutants of RmlA. All the strains were seeded in fresh culture at an initial OD_600_ of 0.1 and grown on a liquid surface at 30°C for 7 days.

To investigate the influence of the RmlA mutations to cell growth, we monitored the RmlA activity in *M. smegmatis* by mass spectrometry analysis. The results showed that the T12D mutant of RmlA has a largely reduced RmlA activity, indicated by the lower level of dTDP-D-Glc as compared to that of the WT strain (Figures 5A,B). To further investigate the influence of the RmlA mutations to cell wall formation, we studied the effect of RmlA mutations to cell wall integrity by applying an acid-fast staining assay. It is reported that *Mycobacteria* contains a large amount of lipid in the cell wall, and once stained, the cell resists discoloration with dilute mineral acids (Wu et al., 2012). Compared with the WT, T12A mutant has a slight decrease in the remaining acid-fast, whereas T12D mutant was almost unable to retain the primary stain after washing with the acid-alcohol decolorizer (Figure 5C). We then further studied the relationship between RmlA activity and biofilm formation, which were strongly implicated in bacterial virulence and in resistance to antibiotics (Ojha et al., 2005). The WT, T12A, and T12D mutants were assayed for formation of the static biofilm (Figure 5D). WT cells initially formed clusters emerging onto the surface, which then steadily spread and eventually covered the entire liquid–air interface. By contrast, the T12A mutant grew slowly and unevenly covered the entire surface after 7 days. Surprisingly, T12D mutant failed to form biofilm with the cell clusters and eventually sank and became submerged in the liquid phase. These results indicate that T12 is important for retaining the proper activity of RmlA and, consequently, in maintaining the cell wall integrity and virulence.

### RmlA Mutants Are Sensitive to External Stimulus and Antibiotics

**FIGURE 6:**
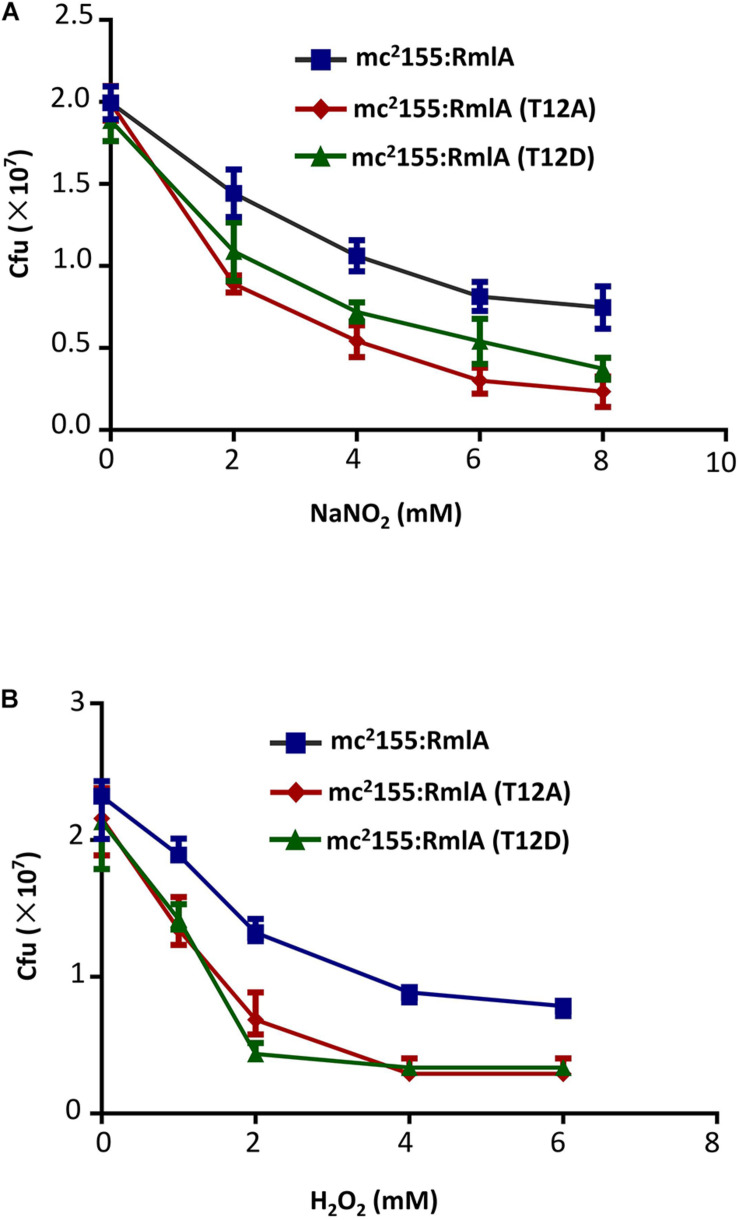
*M. smegmatis* with RmlA mutants is more sensitive to stress. **(A)** Survival of strains after 2 days’ exposure to a series of NaNO_2_ (0, 2, 4, 6, and 8 mM) at pH 5.5. The experiment was performed in triplicates (*n* = 3). Error bars indicate standard error of mean. **(B)** Survival of strains upon exposure to increasing concentrations of H_2_O_2_ (0, 1, 2, 4, and 6 mM) for 12 h. The experiment was performed in triplicates (*n* = 3). Error bars indicate standard error of mean.

Reactive oxygen and nitrogen species are important free radicals for eliminating intracellular microbes in macrophage (Jamaati et al., 2017). To test the virulence of the RmlA mutants to those chemicals, we exposed the three strains (WT, T12A, and T12D) to mildly acidified nitrite, which can kill bacteria through conversion of NO to peroxynitrite. Compared to the WT, the mutants were more fragile to nitrite with significantly reduced cell viability (Figure 6A), especially for T12D. Similar results were also observed when the strains were exposed to H_2_O_2_ as oxidative stress (Figure 6B).

We further investigated the minimal inhibitory concentrations (MICs) of antibiotics related to cell wall formation, such as ethambutol (EMB, an inhibitor for mycolic acid biosynthesis), ampicillin, and vancomycin (Van, an inhibitor for cell wall formation) on the RmlA mutants in *M. smegmatis*. The results showed that vancomycin exhibited a large different in MIC for the mutant strains. The MIC was fourfold lower for T12A and eightfold lower for T12D mutants compared to that of the WT. Meanwhile, there was a slight decrease in the EMB inhibition in the mutant strains (Table 2). Although the MIC of a given compound/drug on *M. smegmatis* (the fast growing mycobacterium) and *M. tuberculosis* (the slower one) may be different, these results proposed a possibility that the potential of RmlA, especially T12, could serve as a target for drug development.

**TABLE 2:** MICs of RmlA mutants to antibiotics.

**MIC (μg/mL)**	**mc^2^155:RmlA (fold change)**	**mc^2^155:RmlA (T12A) (fold change)**	**mc^2^155:RmlA (T12D) (fold change)**
EMB	0.25 (2^10^)	0.125 (2^11^)	0.125 (2^11^)
Vancomycin	2 (2^7^)	0.5 (2^9^)	0.25 (2^10^)
Ampicillin	64 (2^2^)	32 (2^3^)	64 (2^2^)

### The RmlA Mutants Attenuate the Survival of *M. smegmatis* and Promotes Cytokine Production in Macrophage

**FIGURE 7:**
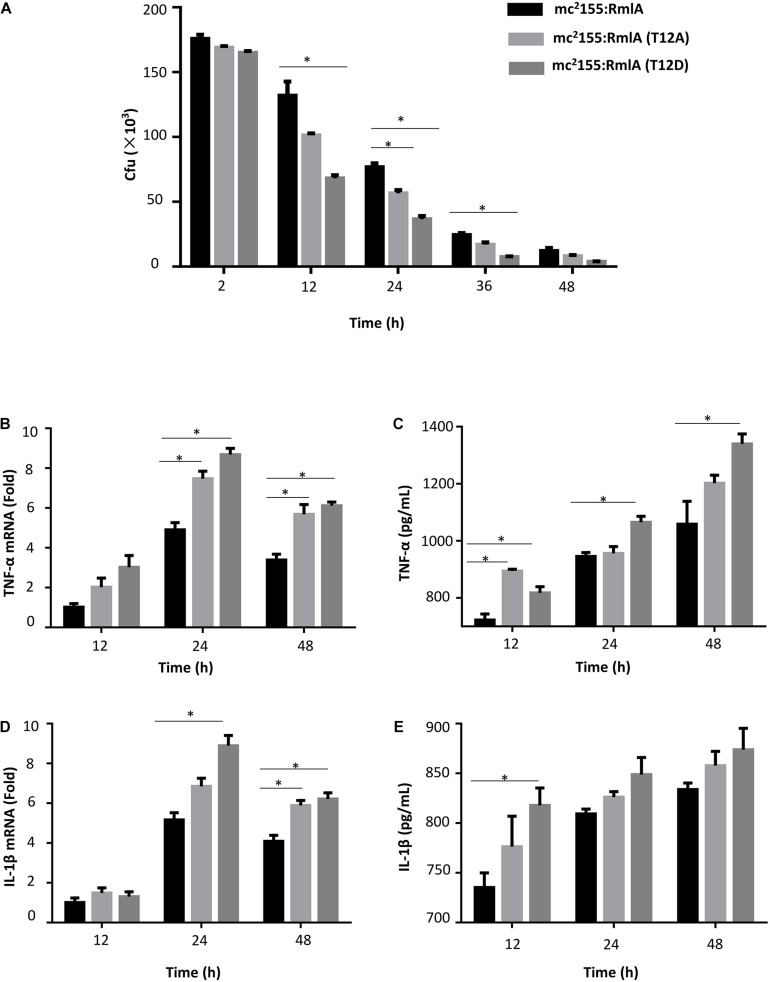
Macrophage infection with *M. smegmatis* RmlA mutants. **(A)** Survival of wild type and mutants of RmlA in primary human monocyte–derived macrophage THP-1 with a series of time points. The experiment was performed in triplicates (*n* = 3). Error bars indicate standard error of mean. Asterisk indicates significant difference (*P* < 0.05). **(B)** Quantitative PCR analysis of TNF-α mRNA level in THP-1 macrophages infected for 12–48 h with wild-type *M. smegmatis* or RmlA mutant *M. smegmatis*. **(C)** ELISA analysis of TNF-α expression in supernatants of THP-1 macrophages for 12–48 h with wild-type *M. smegmatis* or RmlA mutant *M. smegmatis*. **(D)** Quantitative PCR analysis of IL-1β mRNA in THP-1 macrophages infected for 12–48 h with wild-type *M. smegmatis* or RmlA mutant *M. smegmatis*. **(E)** ELISA analysis of IL-1β expression in supernatants of THP-1 macrophages for 12–48 h with wild-type *M. smegmatis* or RmlA mutant *M. smegmatis*.

Inflammatory cytokines such as tumor necrosis factor α (TNF-α) and IL-1β play significant roles in recruiting and activating of the immune system cells in response to *M. tuberculosis* infection (Ribeiro et al., 2016). As followed with the above results have confirmed that RmlA phosphorylation plays important role in bacteria virulence. We speculated that the mutants of RmlA may alter the effects of *M. smegmatis* to macrophages and the host immunologic reaction. To test this, we investigated whether RmlA mutants could affect the expression of inflammatory cytokines in macrophages through analyzing the expression of TNF-α and IL-1β in macrophages infected with RmlA mutants (T12A/T12D) of *M. smegmatis*. There was no significant difference in the survival of the strains between WT and the RmlA mutants within 2 h after infection. While 12 h later, the bacterial viability of the WT was about twofold better than that of the T12D mutants (Figure 7A). Meanwhile, the cytokine expression was increased in cells infected with the RmlA mutants. The results showed that the mRNA levels of TNF-α and IL-1β reached the peak at 24 h after infection with mutants of RmlA (Figures 7B,D); also, the survival of the mutants in macrophage was sharply decreased compared with that of the WT (Figure 7A). Compared with WT, the T12D mutant had a significant increase in the expression of TNF-α (Figure 7C) at various time points, starting from 12 h after infection, whereas the IL-1β expression was slightly improved as compared to that of the WT (Figure 7E). These results indicated that RmlA mutations, especially T12D mutant, attenuate the survival of *Mycobacteria* in macrophage with promoted cytokine production in host cells.

## Discussion

A series of cell wall–related proteins regulated by STPK in *M. tuberculosis* have been reported in recent years (Fiuza et al., 2008; Khan et al., 2010; Le et al., 2016). However, there are only a few reports about the specific substrates contributing to L-rhamnose biosynthesis that are regulated by kinases (Lima et al., 2011; Deng et al., 2014; Wu et al., 2017). Here, we report the significant role of RmlA phosphorylated by STPK, which is linked to *Mycobacteria* cell wall formation and resistance to the environmental stimulation. Through the construction of RmlA T12D mutant in *M. smegmatis*, we demonstrated that the replacement of T12 by Asp residue caused significant activity loss of RmlA. Consequently, a severe cell wall–related phenotypes happened because of the reduction of the cell wall thickness.

Supported by the structure of the RmlA homolog (Alphey et al., 2013) and the phenotypes that we observed for the T12 mutants, it is clear that T12 has a critical role for RmlA activity. The phosphate groups of dTTP lie in an extended position, which interact with the main chain nitrogen atoms of G11, T12, and R13, and also, the γ-phosphate and α-phosphate make a hydrogen bond with T12 and R13, respectively. Accordingly, phosphorylation of threonine could perturb the stability with dTTP. Excluding the T12 phosphorylation site, there are also two other sites, T54 and T197, which could also be phosphorylated, and they are all highly conserved among RmlA homologs (Figure 3A). This study raises a possibility that the observed difference in activity may be associated with the distinct conformations between Thr and Asp in binding with dTTP. Based on the model structure of RmlA in *M. tuberculosis*, it is possible to explain the effect of phosphorylation at T12 on the thymidylyltransferase activity. It is evident that the introduction of a highly charged and bulky phosphate group on T12 would destabilize the hydrophobic region immediately surrounding it (Figure 3B). A T12D mutant generated to mimic the phosphorylated threonine corroborates this as it shows a decrease in the thymidylyltransferase activity (Figure 4B).

L-Rhamnose is a common component of many pathogenic bacteria cell wall. In *M. tuberculosis*, RmlA, an enzyme with dual activity, synthesizes the dTDP-L-rhamnose, which is a precursor for L-rhamnose. Other studies showed that disruption of dTDP-L-rhamnose biosynthesis severely attenuates bacterial fitness and virulence (Rahim et al., 2000; Sassetti et al., 2003; Qu et al., 2007; Carvalho et al., 2015). Interestingly, we found that RmlA T12D mutant caused profound cell growth and reduced the biofilm formation (Figure 5) in *M. smegmatis*. PknB homologs, and other STPKs and phosphatases have been shown to regulate biofilm formation, through involvement of multiple substrates. In *S. mutans*, PknB affects the carolacton acts to disturbing its biofilm viability (Reck et al., 2011). IDH, phosphorylation by PknB in *Staphylococcus aureus*, alters redox status in biofilm formation (Prasad et al., 2015). GroEL, an important chaperone protein in biofilm formation, is phosphorylated by PrkC in *Bacillus anthracis* (Arora et al., 2017). Besides that, a tyrosine phosphatase A (TpbA) also controls biofilm formation in the pathogenic bacterium *P. aeruginosa* (Koveal et al., 2013). And both normoxic and hypoxic growth resulted in substantial decrease in cell viability of the mutant (Figure 6). These results are consistent with the report that *M. tuberculosis rmlA* is an essential gene (Sassetti and Rubin, 2003).

The cell wall of *M. tuberculosis* is an ideal drug target for TB treatment; a series of potent antibiotics that target *M. tuberculosis* cell wall through different mechanisms have already been developed. These antibiotics include cycloserine targeting D-alanine racemase and inhibiting peptidoglycan synthesis (Singh, 2012); isoniazid targeting enoyl-[acyl-carrier-protein] reductase, which inhibits mycolic acid synthesis (Bhat et al., 2010); and ethambutol targeting arabinosyl transferase and inhibiting arabinogalactan biosynthesis (Srivastava et al., 2009), whereas RmlA, as a crucial enzyme for L-rhamnose biosynthesis, is also an ideal target to antitubercular drugs (Ma et al., 2001; Harathi et al., 2016). More importantly, rhamnose biosynthesis pathway is absent in human cells (Maki and Renkonen, 2004). Theoretically, lack of L-rhamnose in humans should eliminate off-target effects, which will lower risks of unwanted side effects (Mistou et al., 2016). We could therefore expect high specificity of the antibiotics targeting *M. tuberculosis* rhamnose synthesis. Increasing knowledge regarding the biosynthesis and function of rhamnose formation pathway could aid the development of new antimicrobial agents. Thus far, several attempts for inhibitor screening for Rml enzymes have been carried out, resulting in only one RmlA inhibitor targeting RmlA of *P. aeruginosa* with some marginal activity against *M. tuberculosis* RmlA (Alphey et al., 2013). Therefore, development of more effective antituberculosis drugs targeting RmlA is still needed, while we have identified T12 as a key site that associates with RmlA’s enzymatic activity, which could prove to be a potential target for drug development.

Taken together, our data showed that PknB phosphorylates RmlA on T12 and further downregulates its thymidylyltransferase activity. The phosphorylation of RmlA causes defects of cell morphology and cell viability of *M. smegmatis* and promotes the sensitivity of *M. smegmatis* to the stress response. When macrophages were infected, the cytokine levels of TNF-α and IL-1β were also increased in the RmlA T12D mutant (Figure 7). These results indicate that RmlA phosphorylated by PknB may play a significant role in regulating cell wall formation to response to the environmental fluctuations/stimulations. This study provides a framework for further investigation on a seemingly important functional linkage between STPKs and the L-rhamnose formation of *M. tuberculosis* and further strengthens the potential of the L-rhamnose biosynthesis pathway, especially RmlA, as an ideal target for developing specific anti-*M. tuberculosis* drugs. We expect that our findings will facilitate drug screening and rational design of *M. tuberculosis* RmlA specific drugs, thus adding a bullet to fight against the ever-growing drug-resistance problem that we face today with TB.

## Data Availability Statement

The raw data supporting the conclusions of this article will be made available by the authors, without undue reservation.

## Author Contributions

S-CT conceived and designed the project. F-LW and DQ performed most of the experiments. XZ and YS contributed reagents or provided laboratory assistance. F-LW and S-CT interpreted results and wrote the manuscript. All authors contributed to the article and approved the submitted version.

## Conflict of Interest

The authors declare that the research was conducted in the absence of any commercial or financial relationships that could be construed as a potential conflict of interest.

## Footnotes

1 https://www.rcsb.org/
