# Supplementary material for: Mycobacterium tuberculosis Thymidylyltransferase RmlA Is Negatively Regulated by Ser/Thr Protein Kinase PknB
Source: Front Microbiol. 2021 Mar 31;12:643951. doi: 10.3389/fmicb.2021.643951 (PMC8044546; doi:10.3389/fmicb.2021.643951)
Supplement: Supplementary file 1 [file Data_Sheet_1.pdf]

Supplemental Figures and Figure legends

Figure S1. Validation of the interaction between PknB with RmlA. (A) Validation of the interaction between PknB with RmlA using BLI *in vitro*. **(B)** Validation of the interaction between PknB with RmlA using Y2H *in vivo*.

Figure S2. Validation of the kinases activities with autophosphorylation. Some of the kinases were purified with His tag in *E. coli*. The kinase reaction was carried out at 37°C for 2 h, and then detected with immune-blotting by anti-ser/thr phosphorylation antibody.

Figure S3. The phosphorylated sites on RmlA by PknB were identified with mass spectrometry.

Figure S4. Construction PknB knock down strain in *M.sm*. (A) Designed sgRNAs targeting the non-template (NT) strand within the open reading frame (ORF) of *pknB*. **(B)** PAM sequences used in the study. **(C)** sgRNAs targeting *pknB* were co-expressed with $dCas9_{Sth1}$ (+ATc). Gene knockdown was quantified by qRT–PCR. The experiment was performed in triplicates (n = 3). Error bars indicate standard error of mean. Asterisk indicates significance difference (P value < 0.05).

Figure S5. The enzymatic activities of RmlA mutants. 4 μg purified RmlA and mutants were added to 50 μL of enzyme reaction buffer (50 mM Tris (pH 7.5), 1 mM dithiothreitol, 5 mM $MgCl_2$, 0.2 mM dTTP, 1 mM D-Glc-1-P), and incubation at 37°C for 1 h.

Figure S6. Construction of rmlA mutants in *M.sm.* **A**, Schematic representation of the homologous recombination. A suicide phasmid pMZ(+) containing *rmlA* and carrying the mutations T12A or T12D, a hyg resistance cassette, the upstream and downstream 800 bp of *rfbA* were used to transduce into *M.sm*. **B**, PCR amplification of *rmlA* sequence. The length of wild type *M.sm* is about 2 kb, while mutation strains which containing hyg cassette, are about 3.5 kb. **C**, Mutations were confirmed by sequencing. **D**, Immunoblotting. Western blot analysis showing the expression level of RmlA with His tag in the crude lysates of the parental strain and the various RmlA mutant strains. **E**, Detection the growth of wild type and mutants of RmlA. All the strains were seed fresh cultures at an initial OD_{600} of 0.1, and the growth was monitored for 2 days at interval of 6 h. The experiment was performed in triplicates (n = 3). Error bars indicate standard error of mean (P value <0.05).

Supplemental Tables

Table S1. Bacteria strains and plasmids used in this study.

Table S2. Primers used in this study.

A

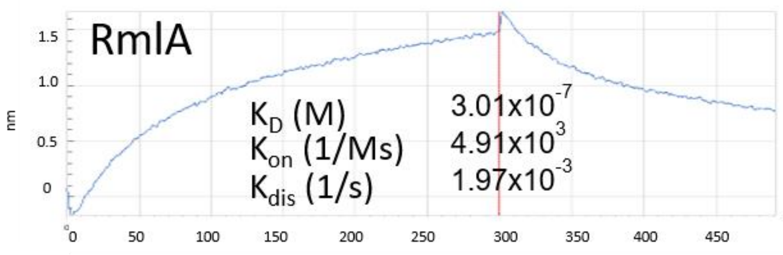

B

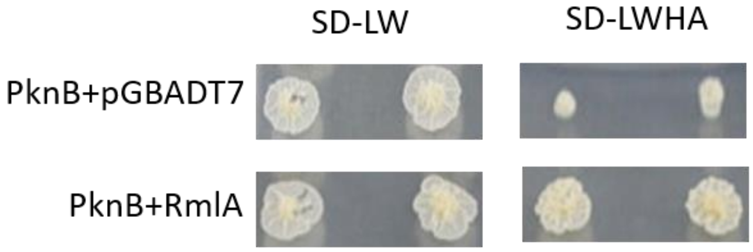

Qu et. al., Figure S1.

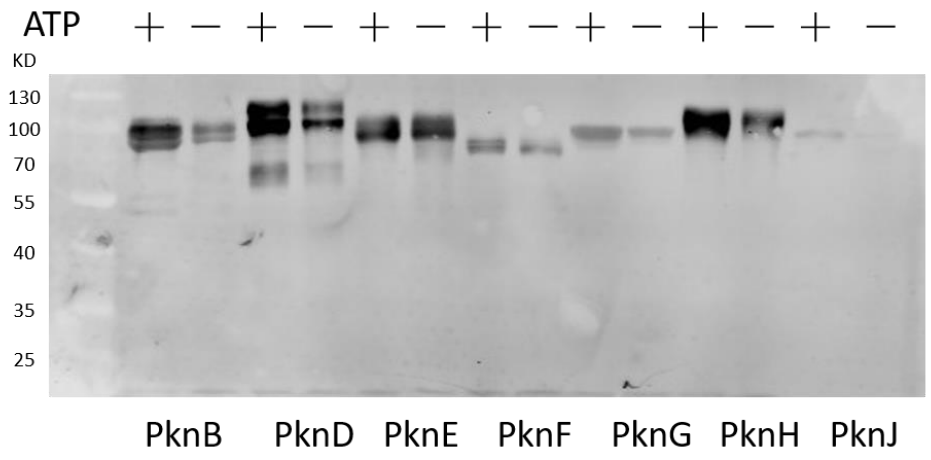

Anti-Ser/Thr phosphorylation antibody

Qu et. al., Figure S2.

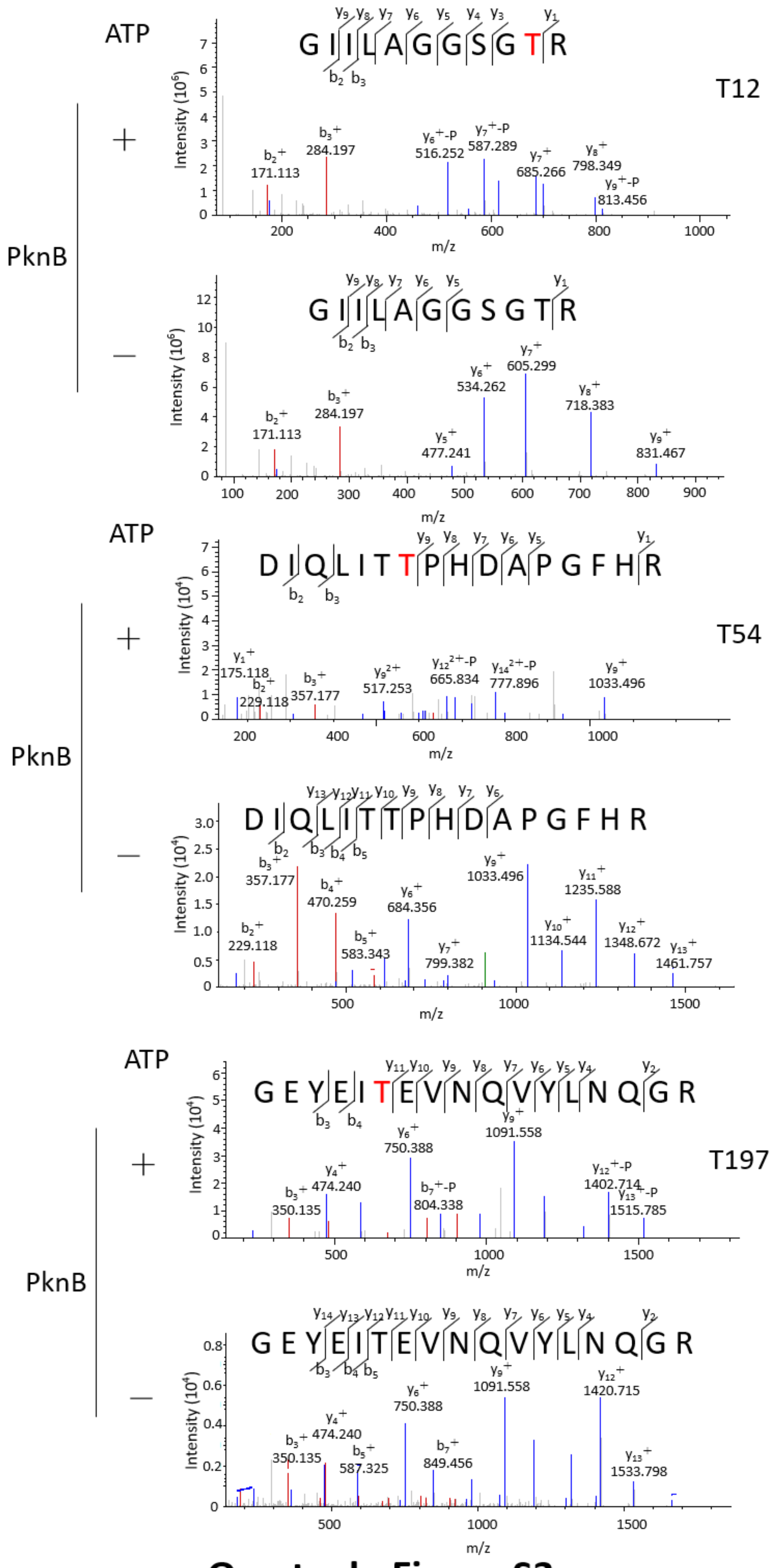

Qu et. al., Figure S3.

A

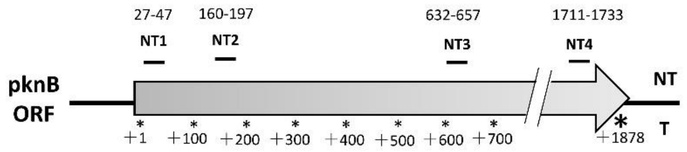

B

NT	PAM
NT1	NNGGAAA
NT2	NNAGAAG
NT3	NNAGGAT
NT4	NNAGAAC

C

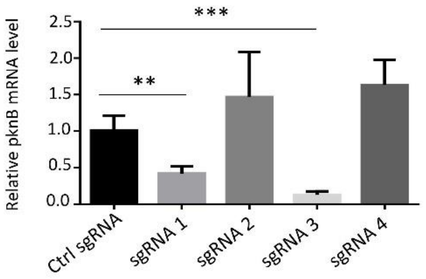

Qu et. al., Figure S4.

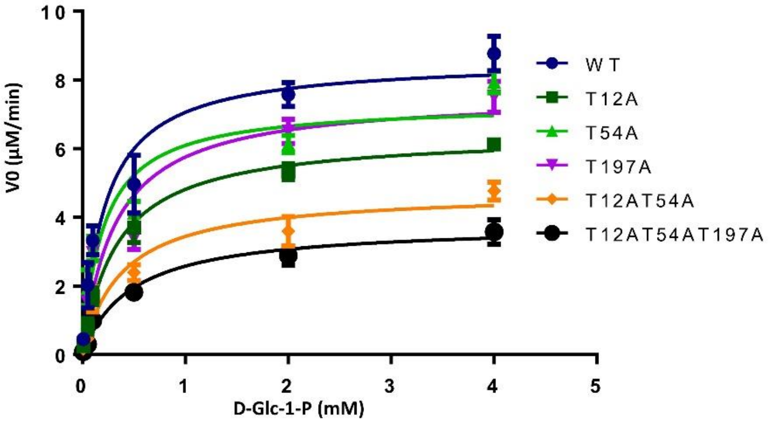

Qu et. al., Figure S5.

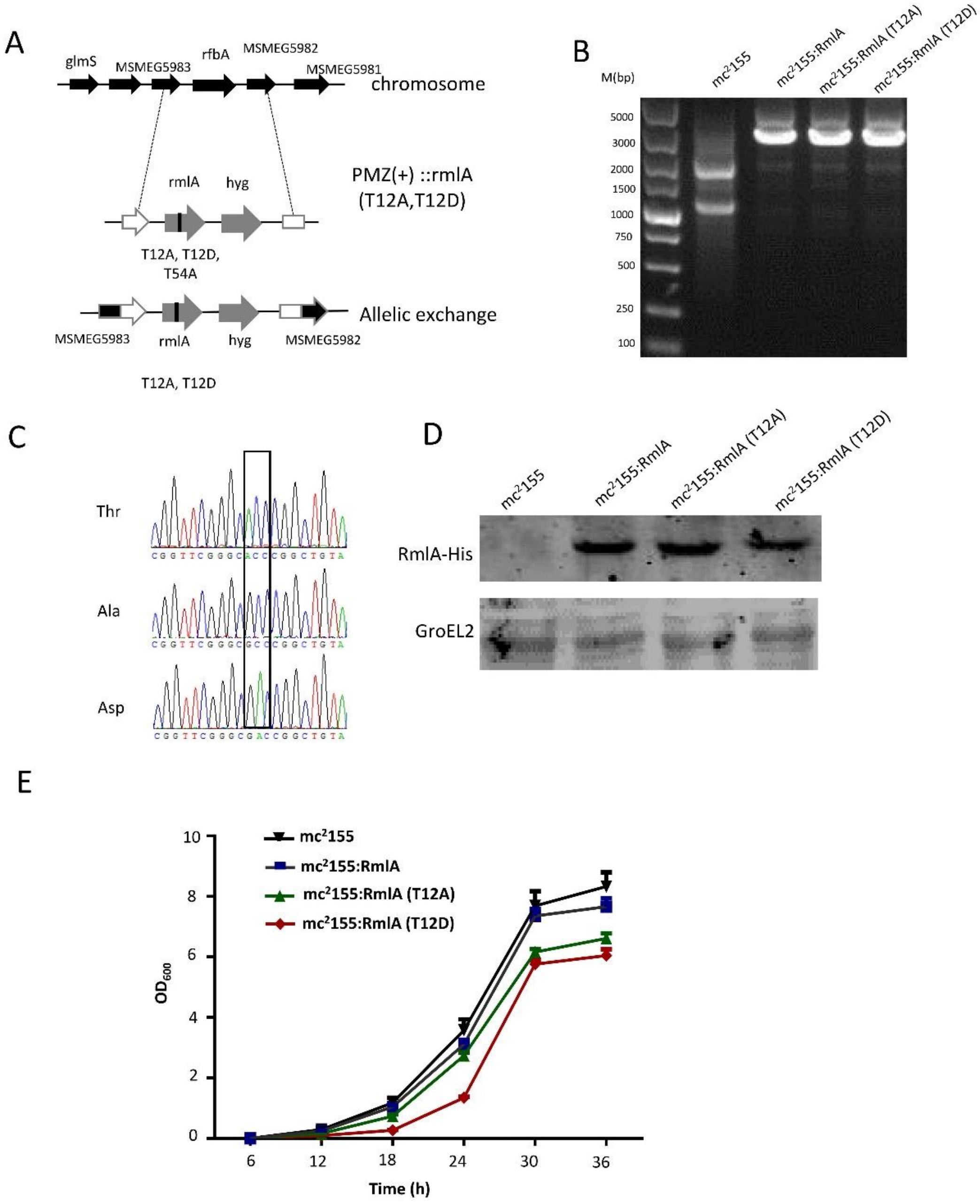

Qu et. al., Figure S6.
